# Factors considered by medical students when formulating their specialty preferences in Japan: findings from a qualitative study

**DOI:** 10.1186/1472-6920-7-31

**Published:** 2007-09-11

**Authors:** Priya Saigal, Yousuke Takemura, Takashi Nishiue, Michael D Fetters

**Affiliations:** 1 University of Michigan Medical School, M4101 MSI Box 0624, 1301 Catherine Road, Ann Arbor, MI 48109, USA; 2 Department of Family Medicine, Mie University School of Medicine, 2-174 Edobashi, Tsu City, Mie, Prefecture, 514-8507, Japan; 3 Sogoshinryoubu, Kansai Medical University, 7-62-306 Ikagakitamachi, Hirakata, Osaka 573-0036, Japan; 4 Department of Family Medicine, University of Michigan, 1018 Fuller St. SPC5708, Ann Arbor, MI 48104-1213, USA

## Abstract

**Background:**

Little research addresses how medical students develop their choice of specialty training in Japan. The purpose of this research was to elucidate factors considered by Japanese medical students when formulating their specialty choice.

**Methods:**

We conducted qualitative interviews with 25 Japanese medical students regarding factors influencing specialty preference and their views on roles of primary versus specialty care. We qualitatively analyzed the data to identify factors students consider when developing specialty preferences, to understand their views about primary and subspecialty care, and to construct models depicting the pathways to specialization.

**Results:**

Students mention factors such as illness in self or close others, respect for family member in the profession, preclinical experiences in the curriculum such as labs and dissection, and aspects of patient care such as the clinical atmosphere, charismatic role models, and doctor-patient communication as influential on their specialty preferences. Participating students could generally distinguish between subspecialty care and primary care, but not primary care and family medicine. Our analysis yields a "Two Career" model depicting how medical graduates can first train for hospital-based specialty practice, and then switch to mixed primary/specialty care outpatient practice years later without any requirement for systematic training in principles of primary care practice.

**Conclusion:**

Preclinical and clinical experiences as well as role models are reported by Japanese students as influential factors when formulating their specialty preferences. Student understanding of family medicine as a discipline is low in Japan. Students with ultimate aspirations to practice outpatient primary care medicine do not need to commit to systematic primary care training after graduation. The Two Career model of specialization leaves the door open for medical graduates to enter primary care practice at anytime regardless of post-graduate residency training choice.

## Background

During preclinical and clinical experiences medical students construct their professional identity through a process of medical socialization [1]. Within this socialization context focused on acquisition of new knowledge and skills, interactions with other medical students, health professionals, and patients, students construct their professional identity grounded in principles of the biomedical model [2]. Cultural and societal values also influence future physicians, particularly through student interactions with family, friends, and physicians [3]. The process of resolving conflicting views from societal constructs and self-realizations shape student views on the type of specialist they want to become, for example, primary care versus specialty care.

While a body of literature from around the world including countries such as the United States (U.S.) [3], Australia [4], New Zealand [5], Canada [6], and China [7], address factors affecting medical student specialty choice, there is little data on influences on Japanese medical students [8]. While the international studies suggest that there is a high demand for family practitioners [6,8], it is often difficult to recruit medical students into family medicine [6]. This body of previous research reinforces the idea that experiences during clinical training have an important impact on medical students' preferences for specialty selection; and interventions to influence specialty preference should target this period of training [4,6,7]. Given growing interest in family medicine by medical students and resident physicians in Japan, examination of factors affecting specialty preference bears great importance especially since the Japanese Government has yet to recognize family medicine as a specialty. Since family medicine is not a board certified specialty, there is no official count as to the number of programs in primary care. However, the Japanese Academy of Family Medicine is working to certify residency programs through the specialty society [9]. Systematically trained primary care physicians are clearly needed given Japan's rapidly aging population and high prevalence of chronic ailments [10]. The bias to train in non-generalist, subspecialty care in Japan's pluralistic medical system is rooted in unique historical and social traditions [11] and likely contributes to few medical students entering primary care after graduation [12]. In Japan, few institutions systematically train primary care physicians [13]. For Japanese students interested in primary care, training options at graduation remain limited. In addition to the lack of recognition of family medicine as a discipline, there is no requirement for systematic, general training or certification to practice primary care medicine.

For the sake of clarity, it is worth noting that the term primary care and family medicine have distinct meanings in different countries. For example, the U.S. Institute of Medicine (IOM) defines primary care by its function stating it is "the provision of integrated, accessible health care services by clinicians who are accountable for addressing a large majority of personal health needs, developing a sustained partnership with patients, and practicing in the context of family and community". [14]. By this definition, primary care specialties include family medicine, general internal medicine, pediatrics, and obstetrics/gynecology. The United Kingdom's Department of Health defines primary care as all those health services provided outside the hospital, commonly provided by general practitioners or family doctors [15]. Since Japan has few physicians trained in the delivery of primary care, and lacks recognition of the discipline of family medicine, here we use the term primary care to refer to the function as stated by the IOM. We distinguish between physicians who provide primary care as a function, and those trained in family medicine, the discipline. For developing family medicine as a discipline in Japan, determining how students and physicians understand the role and definition of primary care and family medicine is needed.

Several comments about the Japanese medical education system and clinical practice provide necessary context for the current discussion. Two recent articles by Kozu and Teo provide excellent updates about medical education and training in Japan [16,17]. Briefly, there are 79 medical schools in Japan, and about 7,900 students graduate from medical school each year (about 100 per medical school). Starting in 2004, virtually all students who intend to engage in patient care are required to complete two years of preliminary training through a new matching system. After two years of training, students may enter any specialty program of their choice, but must arrange this on their own without benefit of a matching system. These post-graduate training programs generally last 4–6 years. The number of positions in any given program is fluid; from a program perspective, the more that match, the better for the program as there is a bigger labor force. About 100 of 2,200 post-graduate, medical education training programs consider themselves "General Medicine" (*sôgôshinryô*) programs [17], though many of these function as hospital triage units, and do not have a true mission of training primary care doctors. Very few have truly embraced family medicine as their guiding discipline [18]. In a 2005 survey of second year residents, 3298 (86.6%) indicated their specialty training intent. Only 0.8% indicated an intent to specialize in general medicine, a very small number compared with those intending to specialize in other disciplines: internal medicine (14.6%), general surgery (8.9%), pediatrics (7.5%), OB/GYN (4.9%); surgical subspecialty (34.3%), medical subspecialty (24.9%), and other (3%) [19].

Despite a lack of family medicine/primary care-focused training programs, it has been estimated that of Japan's 230,000 doctors about 60,000 are community-based and essentially function as general practitioners without systematic training in primary care [8]. While the government recently started experimentation with encouraging private practitioners to admit to open access community hospitals (*kaihougata byouin*), private practitioners primarily provide care in their own outpatient clinic or small hospitals [13], and do not continue many of the intensive, more risky procedures, like cardiac catheterizations or surgical procedures, learned during their subspecialty training. Brain surgeons in private practice, are unlikely to bore any skull holes in their office. Regardless of specialty, Japanese physicians are likely to earn a significantly higher income as a private practitioner than working as a hospital employee [13]. Finally, there is no restriction in terms of practice spectrum in Japan. A private practitioner can post a billboard advertising the content of medical practice chosen without legal restrictions as long as the specialty advertised conforms to one from the official specialty names list [20].

Understanding of students' views about career choice and training could help inform establishment of primary care training programs in Japan. Although Ohtaki et al have conducted research on specialty choice [8] in Japan, their study's close-ended written questionnaire format limited the breadth and depth of insight into the Japanese students' thought processes about their specialty preference. Here, we examine factors Japanese medical students consider while formulating a preference to pursue a primary care versus subspecialty care career. In addition, we examine their understanding of family medicine, primary care, and subspecialty practice. These data may allow Japanese medical educators to direct future interventions towards improvements in recruitment and training of primary care providers.

## Methods

### Design

This qualitative project was conducted using semi-structured interviews with medical students, informal interviews with academic faculty in the host institution, and field notes. Data collection spanned the months of June to July 2004. We used qualitative methodology in this study as an initial step of research about factors influencing medical specialty preference in Japan, and to aid hypothesis generation for subsequent research [21]. Interviews typically lasted approximately 30 minutes and focused on how the medical school curriculum and environment influences student career preferences for primary care versus specialty care. The University of Michigan Institutional Review Board reviewed and approved this investigation. The study was judged as exempt from requirement for Institutional Review Board approval and monitoring at Mie University.

### Participant recruitment and sampling

Undergraduate medical education typically involves a six-year college program in Japan with the first two years involving general coursework. We thus sampled Japanese medical students in their third through sixth years of training as these most closely approximate the four years of U.S. medical undergraduate education. Each class has about 100 students. We designate 3^rd ^and 4^th ^year students as preclinical, 5^th ^and 6^th ^year students as clinical. We recruited participants using posted notices and email announcements. One of us (PS), who was trained by one of the senior investigators (MDF), interviewed all students who volunteered to participate; no subjects were excluded.

### Consent and data collection

Prior to the interview, all participants reviewed and signed a Japanese-English bilingual consent form, and completed a demographics questionnaire. During the interview, participants first received the list of open-ended interview questions written in English and Japanese, as participants preferred this style. (Appendix 1) One of us (PS) conducted the interviews in English. A bilingual Japanese physician was available to interpret during the interviews, though some students preferred speaking in English. All interviews were digitally audio taped using Cool Edit 96^® ^software and stored as electronic wave files on CD's.

### Data analysis

The study coordinator (PS), using a standardized protocol, selectively transcribed the English sections of the audio taped recordings of interviews. Selective transcription involves transcribing only the information deemed relevant to research topic [22]. Using this methodology, the selective transcripts omitted only those portions of conversations which were irrelevant to the interview process, i.e. opening sentences and warm-up conversations about the weather, day, etc. A native Japanese speaker transcribed all Japanese language sections of the interviews. A bilingual researcher reviewed each transcript for accuracy of speakers' actual words and accurate translation from Japanese to English. Though Japanese spoken by the interview subject was frequently summarized when interpreted into English, the content was conceptually accurate.

Based on multiple readings of transcripts and notes, themes addressing student preferences in specialty training and understanding of primary care were iteratively identified by the primary analysis team (PS and MDF). From the informal discussions with faculty members and reflections about our previously identified biases (procedure for understanding how each researcher's biases could influence the research) [23] we identified for comparison faculty perceptions about students' attitudes. As a final step, we developed a conceptual model depicting the pathways to a career in primary care in Japan. For comparison purposes, we developed a parallel conceptual model to depict training pathways in the U.S. The U.S. model was derived based on the authors' knowledge of the matching system, reimbursement for graduate medical education, and the requirement of board certification to make a claim of being a specialist in a specific discipline.

We conducted member checking (a process of verifying the accuracy of the results) [22] by discussing, in person, the primary findings with six of the students and informal discussions with four other students from a second medical program. We iteratively reviewed the themes with previously interviewed students in order to verify the findings, elicit missing details, and explore for evidence contradicting our findings. Additionally, we had in-depth discussions with several Japanese and American physicians who have expertise in the health care systems of both the United States and Japan to validate the findings. These discussions allowed us to develop a context in which to interpret the data collected, with a better overall understanding of local culture, norms, and medical practices.

## Results

### Demographics

Twenty-five students participated (17 male, 8 female); this roughly reflects the gender distribution at Mie University Medical School. Eight preclinical and 17 clinical students participated. Student age ranged from 21 to 35. Approximately one third of the students have physicians in their immediate family, and about one quarter have either physicians in their extended family or siblings in medical school. About half of the participants (n = 13; 9 males, 4 females; 6 preclinical, 7 clinical) preferred primary care specialization at the time of the study.

### Motivations for entering the medical profession

Students provided a range of reasons for entering the medical profession (Table 1). Several students reported that personal experiences with illness or witnessing their loved ones suffer from illness influenced their interest in medicine as a career and often their specialty choice. For example, one clinical male student reported, "...*I have no doctors in my family, but I have suffered from my own disease. I suffered from neurosis, family relationship problems*." Eleven of the twenty-five students reported that they changed their specialty choice during medical school. Some students reported that the academic experience during their preclinical training, lab research, basic science course work, or extracurricular activity affected their specialty choice.

**Table 1:** Influence of personal, preclinical and clinical experiences on Japanese medical student specialty preferences

**Personal experience**	• "... *I had a friend who had anorexia, and she was very sad, so I wanted to help her... I want to be a psychiatric doctor for children...*" clinical female student
	• "... *My father is a neurosurgeon, and my grandfather and uncles are physicians... I often injured myself when I was a kid. My father cured the injuries. I respect him... My father was very busy when I was a child, but I saw him working in the hospital, and I respected him a lot*... " clinical male student
	• "... *My father is an endocrinologist. Many patients love my father, because some patients told my father that I feel I get well by looking at your face... My father is respected by many patients, so I respect my father*." preclinical male student
**Pre-clinical experiences**	• "... *I heard many people's opinion about my being a surgeon as a female. They say it is very difficult for women. In Japan, women when they marry, they have to do more home things and child care*."
	• *"...I want to use my hands and I want to help people with my hands... I have always wanted to be a surgeon, and I enjoyed dissecting rats in the laboratory and anatomy class during 1^*st *^and 2^*nd *^ year... " *preclinical male student
	• "... *First of all, I have only had classroom lectures about what primary care is, and how is it done in the community. I want to actually see it with my own eyes, feel it, and learn it*." clinical female student

**Clinical experiences**	• *"... I experienced patient contact only during primary care so far in my clinical clerkships." *clinical female student
	• *"... Now I have the recognition of the very complex relationship of society with hospitals and doctors... I enjoyed my off campus experiences in social medicine and rural health most." *clinical male student
	• *"... Well, yeah, with regard to what educational processes had an influence, it has been since the spring of fifth year. Yeah, what especially left an impression was psychiatry... for me, at least right now. (Why is that so?) [Its] very impressive. Communication with patients is impressive, they take lots of time. I think you can do a lot." *clinical male student
	• *"... Originally I wanted to be a generalist, but Dr. K in the department of general surgery has made me interested in surgery as I much admire him and his worldwide fame." *Clinical male student

### Participant reports of factors that affected their specialty preference

Students' evaluations of their overall experience in providing or observing patient care during clinical rotations appear to weigh heavily. Students report several factors associated with their clinical clerkships as affecting their specialty choice: opportunity to have direct patient contact, length of clinical exposure, quality of interactions with faculty physicians, personality of physicians, presence of a physician role model or mentor, and the overall atmosphere within departments in health care settings. A clinical female student confided, *"... Before I wanted to be a surgeon, because I thought they were cool... Now I want to be in pediatrics because I really enjoyed the atmosphere during my 5^*th *^year rotation." *Table 1 provides additional quotes of medical students at Mie University who found particular aspects of the clinical environment encouraging or discouraging.

### Students' views on primary care, subspecialty care and family medicine

Medical student responses regarding the roles of physicians in Japan provide insight into the rationale for making their specialty choice and their views of physicians' social roles. Table 2 summarizes words and phrases subjects used to distinguish between different areas of specialization. For example, one student stated:

**Table 2:** Student views regarding roles of primary care, subspecialty and family physicians

	**Role of primary care physicians**	**Role of subspecialists**	**Role of family physicians**
**Location**	• Community based	• University based practice	• Solo practice in neighborhood setting • Private practice in the community • Cannot be properly practiced in hospital setting
**Scope**	• Primary consultation before seeing specialists • Focus on care, not cure • Common disease, easy to treat • Psychosocial concerns • Long term care	• Temporary care • Focused on specific body part, not whole • Complex disease treated with difficult procedures	• First contact in coordinated patient care • Long term care of patients and their diseases • Home visits • Continuity of care • Psychosocial skills • Mental care provision • Home doctor • Focus on prevention
**Patient relationship**	• Good doctor-patient relationship • Treat the whole body	• Focus on cure, not care • Treat disease, not person • Evidence based	• Patient oriented medicine • Sees patient as a whole • Care about whole family
**Expertise**	• Broad knowledge • Home visits are important • Care for entire family • Focus on prevention, triage, and medical interviews	• Expert knowledge • Many specialists also provide primary care services • Surgeons are expert specialists	• Broad knowledge • Home care • Care for entire family • Prevention of disease in the community

*Differences in specialist and primary care???...Well, you can see everything, yeah, you can see all patients. And...another important thing... being accessible, close in distance, and also close emotionally, by being close by. Well, and without it being a big affair, getting advice. Um, especially, [Japan] is going to be an aged society, and there are home care visits... Yeah, there are going to be many chronic problems, so the doctor who is close by is more important. I am not really sure, but, a family physician, (provides) home care, right in that house. And also, probably what they do is centered around the home. Primary care has that too, but, rather the hospital, yeah the hospital, the hospital and the family, its both. It seems that primary care is broader, but...it seems as though what they are doing doesn't differ much... Yeah, that's the overall picture*.(clinical male student)

From this and other statements, it appears students have some understanding, though incomplete about differences in primary care, subspecialty care and family medicine. This comes as little surprise since Japanese society does not define family medicine as a distinct specialty, and subspecialty-trained physicians ultimately serve as primary care providers.

The blur between primary, subspecialty and family medicine appears to be rooted in the reality of physicians who provide primary care. Students tend to view primary care as a second career in medicine that follows an initial stepping-stone of 10–15 years of training and working as a subspecialist. One student stated:

"I will visit the rural clinic in the village. The physician I will visit in the village was a pediatrician in the hospital, and now he is a family physician in the community."

Another student stated:

"*Well...(pause) as for my thoughts right now, I am leaning towards emergency medicine after graduation... I want to achieve a sufficient level of competency, then, for example 10 years later, when it becomes physically burdensome, well, I think I will want to go into primary care*."

Students can see and project for themselves, a first career in hospital-based specialty care, then changing gears later for a second career in outpatient primary care practice.

### One versus two career model of specialty practice

**Figure 1:**
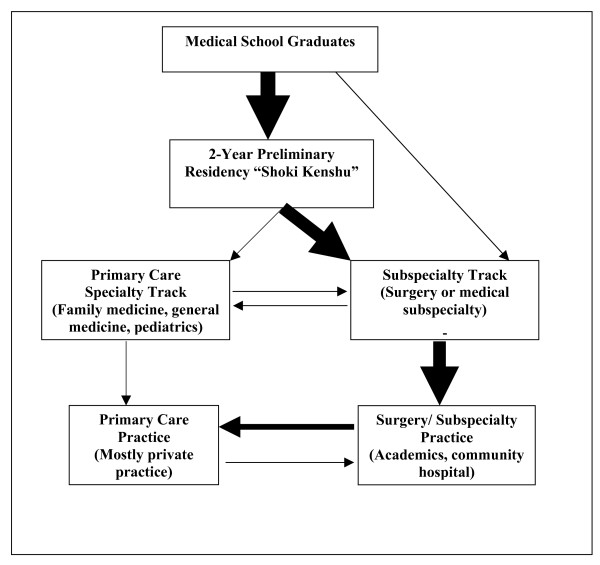
Two-career specialty model of specialization in Japan depicting the flow of medical graduates into primary care and subspecialty care.

**Figure 2:**
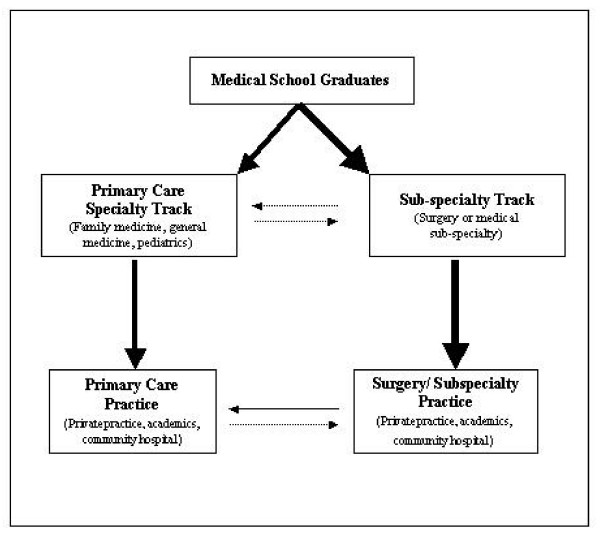
One-career model of medical specialization in United States depicting the flow of medical graduates into primary care and subspecialty care.

These findings from the interviews with medical students and discussions with multiple physicians on faculty, suggest the pathways to primary care practice differs greatly in Japan and the U.S. (Figures 1 and 2). In these models, the thickness of the arrows approximates the percentage of individuals who choose to pursue that pathway, e.g., a thick arrow reflects a higher percentage of graduates. Dotted lines further denote infrequent activity.

Figure 1 represents the flow of medical graduates into practice in Japan, and illustrates the Two Career model of medical specialization. Almost all the graduates must complete a two-year preliminary residency before they can continue to their chosen tracks; however there is a small percentage that foregoes preliminary residency training and enters subspecialty training directly. Thereafter, the vast majority will continue in subspecialty training, and a few will pursue primary care training. These numbers are also reflective of the postgraduate training opportunities that are available to recent graduates, as there are very few primary care training programs in Japan.

The unique aspect of the Two Career model is that 10–15 years after graduation, a large number of physicians transition from hospital-based subspecialist practice into outpatient-based subspecialty/primary care practice. Without open access hospitals, subspecialty private practice becomes greatly restricted as it becomes infeasible to conduct medical and surgical procedures that require hospital support. This constrains practitioners to a limited range of their subspecialty skills learned in residency. They become default primary care practitioners [8]. They must include first contact and accessible care for common problems that characterizes primary care in their "second career". The content of the second career in office-based practice includes clinical content of their subspecialization to the extent that it can be practiced in an outpatient setting, as well as generalist care for common problems. While trained for the former, they typically assume the generalist role without any mandatory or systematic training for primary care. Hence, this pathway reflects a Two Career model. Many physicians initially have a hospital-based subspecialty career, usually in academic or community hospitals, and later take on a mixed, office-based hybrid subspecialty and primary care career.

In the U.S. the vast majority of physicians in primary care practice traverse systematic residency training in primary care before going into primary care practice. Figure 2 depicts the flow of medical graduates into practice as a One Career model of medical specialization. The majority of recent medical graduates choose to pursue the subspecialty track after medical school, whereas a minority will opt to pursue training in a primary care discipline. There is minimal transition between these two pathways during graduate training; though there are some subspecialists who choose to transition their clinical practice into primary care. Since the government limits the number of years salary will be paid for post-graduate training, it is difficult for U.S. medical residents to transfer from one discipline to another, and relatively few do. Moreover, completion of residency training is required for board certification for virtually all disciplines. Consequently, it is difficult to practice outside of one's area of specialization after being certified. In short, subspecialty trained physicians will continue to practice subspecialty care, e.g., urology, even if they forego hospital-based practice. Most board certified physicians will continue to practice within their certified specialty for their entire career.

## Discussion

We find many factors reported as influential by Japanese medical students to be consistent with data from previous international studies [5,7,24,25]. Both personal and academic (preclinical and clinical) experiences are important in shaping students' perceptions of specialties and their personal choices. Zarkovic et al found physician role models to play an important role in the specialty decision-making process for medical students in New Zealand [5]. In our study, many students stated that they were interested in particular fields based on positive interaction(s) with patients and physicians within that specialty. Similarly, some students were discouraged to consider medical specialties after they had a negative experience on the wards or interaction with a faculty member. Harris et al found Australian medical students also to be strongly affected by both intrinsic factors and their contact with the work environment early in their medical training [4]. These factors appear to hold, despite differences in the cultural values and health care systems of several countries for which there are data on specialty choice and preferences.

Despite the similarities, the "Two Career Model" illustrates that career pathways medical students consider in Japan look very different. Japanese medical students receive their education in an environment where it is widely held that hospital-based training prepares one for both subspecialty care and for outpatient practice of primary care. Though landmark data about Japan have recently been published [26], subspecialists generally lack an understanding of the ecology of medical care and the different skill sets and knowledged needed to practice [27]. Private practitioners consider (mistakenly in our view) that their subspecialty training is adequate to be clinically competent for practicing high quality primary care.

Physicians receive minimal outpatient-oriented general medicine training during residency, even with the recent requirement of a two-year rotating internship, as virtually the entirety is spent in inpatient care. Moreover, there are no standards for "re-training" residency programs designed to systematically prepare such physicians for primary care practice. Broader skills are often acquired by moonlighting in private practice offices, or arranging time in departments that offer clinical opportunities to learn knowledge and skills they believe necessary to make the transition to outpatient practice. For example, a residency trained breast surgeon may spend a few months working in an endocrinology ward. An urologist may work in a dermatology ward or clinic before going into practice. This kind of supplementary cross training is ubiquitous in Japan, and possible for two reasons. First, experienced, non-resident physicians can easily make personal arrangements to work in a specific hospital department as a staff member while essentially doing work as a resident. The hospital, not the government, pays their salary. Second, there are rather loose requirements for board certification in Japan. While there are board certification exams for some specialties, physicians can affiliate with specialty societies with little more than paying the membership fees. Credentialing procedures are virtually non-existent, so movement from one department to another or even one institution to another is very easy after personal negotiations are completed.

The well-established "Two Career Model" raises serious implications as a barrier for establishing family medicine and other primary care residencies in Japan. The Japan Medical Association (JMA), whose membership has largely followed the "Two Career Model" into private practice, holds that all physicians in Japan are qualified to provide primary care. Accepting family medicine could raise questions about the legitimacy of physicians who enter primary care through the "Two Career Model". Moreover, decades ago the Ministry of Health, Labour and Welfare proposed adoption of family medicine as a mechanism to limit health care costs, and this generated vigorous opposition to establishment of family medicine by the JMA that persists today as it was viewed a means to limit income [28]. A full treatise on the reasons for and history of the opposition is beyond this paper, but suffice it to say that opposition ranges from ambivalent to extreme.

Our data suggest that Japanese medical students at minimum are aware of the "Two Career Model" pathway. Consequently, medical students interested in primary care can initially train in a subspecialty, knowing that the prevalent societal norm allows them to practice primary care later in their career without questions about their qualifications. Coupled with the paucity of well-developed family medicine and other primary care training programs, it comes as little surprise that many Japanese students will avoid postgraduate training in primary care.

We found that faculty members believe that few students are interested in primary care, since few students choose primary care specialties for their post-graduate training. Our data suggest that this may be a misperception as many of these students expressed an interest in primary care specialization. Unfortunately, there are few resources or residency programs that will prepare them for primary care in a one-career pathway. The direct path is risky since neither the Japanese government nor the JMA recognizes family medicine. Finally, the Two Career model of specialization includes a well-traveled pathway through initial training and hospital-based work as a subspecialist followed by a career in primary care medicine. Students can subspecialize without limiting their option to practice primary care.

Our results further imply that improving the quality of primary care and recruiting medical students to pursue primary care specialization may require training programs designed for the One Career and Two Career Models. Unlike the UK where virtually all outpatient doctors are considered primary care doctors as they have received training in primary care, Japanese outpatient doctors have virtually no training in primary care[8] While the number of programs specializing in primary care training is still limited, the single career pathway of post-graduate training in family medicine is establishing a foothold despite opposition from the JMA and ambivalence on the part of the Japanese government. Since practicing physicians do influence some students' choice of specialization, routinely exposing students to primary care physicians during clinical years at medical schools is needed. More family medicine-based training programs are needed as well. Interestingly, second career models of re-training are under development. One innovative program at Jichi Medical School, Department of Community and Family Medicine developed a two-year, re-training program for individuals with at least five years clinical experience who are ready to leave hospital-based specialty care and enter the primary care world [29]. A two-fold strategy addressing the ''One Career'' and ''Two Career Models'' could facilitate the establishment of family medicine training programs in Japan.

This study has potential limitations. First, the host Department at Mie University takes a leadership role in establishment of family medicine in Japan. Students volunteering for the study might have known the departmental bias towards primary care. Second, for feasibility issues, namely, time and limited budget, the data were collected from a limited sample of 25 students (about 6% of eligible 3^rd ^to 6^th ^year students), and these data were not collected with the intent of being generalizable to all medical students. Still, we are doubtful there would be substantively different issues even if recruiting a larger sample had been feasible.

Future research should examine the relative importance of the aforementioned factors' influence on primary care and specialty choice for Japanese medical students. Moreover, research is needed on popularity and effectiveness of re-training programs for physicians who initially train as subspecialists and desire a career in community-based, mixed primary care/subspecialty practice. Future research may also shed light on the relevance of the "Two Career Model" in other countries along with the implications for primary care training and practice.

## Conclusion

Medical students participating in this study report preclinical and clinical experiences, as well as role models, to be influential factors on the formulation of their specialty preferences. Most students have at best, a rudimentary understanding of family medicine as a discipline. In Japan, students interested in practicing outpatient primary care medicine can specialize in virtually any other discipline initially after graduation, but ultimately still practice primary care without any systematic primary care training. The Two Career model of specialization permits medical graduates to enter primary care practice at anytime regardless of post-graduate residency training choice or clinical practice.

## Competing interests

The author(s) declare that they have no competing interests.

## Authors' contributions

All authors contributed to the writing of the manuscript and reviewed and approved the final submitted draft. PS and MDF were the principal authors and they designed the interview instrument. PS conducted the interviews and collected the data at Mie University. YT critically reviewed the interview guide, participated in the design of the study, and coordinated the recruitment of interview subjects at Mie University School of Medicine. TN contributed to the study design, participated in reviewing the accuracy of the Japanese interpretation, and helped to draft the manuscript.

## Appendix 1. Interview questions on factors influencing Japanese medical student choices for specialty training

1. Do you have any immediate family member who is a physician? How has this influenced your decision to pursue medicine and your fields of interest?

2. What specialty would you like to go into as a doctor? What fields do you not want to go into? What kind of work interests you (surgery, procedural, consultation, lifestyle, etc.)? How have you reached this position?

3. How have your views about your preferred medical field changed through your medical education? What elements within the curriculum have influenced your current views? How has the medical school environment, classroom, non-classroom social and cultural events or activities influenced your views? Have you had formal clinical training in family medicine?

4. What is your understanding of primary care? What does primary care involve for doctors? What is specialty care? What does specialty care involve? What is the typical work for a primary care and specialist doctor?

5. What are your options in receiving clinical training in primary care? How did you learn about these options?

## Pre-publication history

The pre-publication history for this paper can be accessed here:


